# Effect of emotional arousal on inter-temporal decision-making: an fMRI study

**DOI:** 10.1186/s40101-015-0047-5

**Published:** 2015-03-07

**Authors:** Jin-Hun Sohn, Hyo-Eun Kim, Sunju Sohn, Ji-Woo Seok, Damee Choi, Shigeki Watanuki

**Affiliations:** Department of Psychology, Brain Research Institute, Chungnam National University, Koong-dong 220, Daejeon, Yusung-gu 305-764 South Korea; School of Design, Graduate School of Design, Kyushu University, Shiobaru, Minami-ku, Fukuoka, 815-8540 Japan; Department of Social Welfare, Cheongju University, Cheongju-si, Chungbuk 363-764 South Korea; Department of Kansei Science, Kyushu University, Shiobaru, Minami-ku, Fukuoka, 815-8540 Japan

**Keywords:** Emotion, Arousal, Impulsivity, Delay-discounting task, Decision-making, fMRI

## Abstract

**Background:**

Previous research has shown that emotion can significantly impact decision-making in humans. The current study examined whether or not and how situationally induced emotion influences people to make inter-temporal choices.

**Methods:**

Affective pictures were used as experiment stimuli to provoke emotion, immediately followed by subjects’ performance of a delay-discounting task to measure impulsivity during functional magnetic resonance imaging.

**Results:**

Results demonstrate a subsequent process of increased impulsive decision-making following a prior exposure to both high positive and negative arousal stimuli, compared to the experiment subjects’ experiences with neutral stimuli. Findings indicate that increased impulsive decision-making behaviors can occur with high arousal and can be characterized by decreased activities in the cognitive control regions such as prefronto-parietal regions.

**Conclusions:**

These results suggest that ‘stabilization of high emotional arousal’ may facilitate a reduction of impulsive decision-making and implementation of longer term goals.

## Background

In a wide range of decision-making contexts, individual choices are said to be the outcomes of a competitive interaction between the following two systems, also known as the dual-system model: affective system and deliberative system [1-4]. The affective system is primarily driven by visceral factors such as negative emotions (for example, fear) or drive states (for example, sexual desire); therefore, it relates to individuals’ myopic and impulsive choices due to people having limitations as to predicting and/or evaluating beyond the present moment. The deliberative system, on the other hand, assesses options with a forward-looking, goal-oriented perspective; therefore, it subserves the executive functions, such as planning, thinking ahead, or self-control.

Based on the dual-system model, the deliberative system in humans has the ability to impose regulatory control over impulsiveness and risky behavior under the circumstances in which the affective system is not highly activated. According to the dual-system model, the deliberative processes are mainly associated with the outer part of the brain, including the anterior and dorsolateral regions of the prefrontal cortex and parietal cortex, while affective processes are associated with the inner part of the brain, including the limbic and paralimbic areas of the brain, amygdala, ventral striatum (VS), and insular and medial prefrontal cortex (mPFC) [2,3]. Therefore, under the influence of emotional arousal, the affective system elevates to a sufficient level to activate and weaken the regulatory functions or effectiveness of the deliberative system [3].

Numerous behavioral studies have also suggested that situationally induced emotion can also significantly impact inter-temporal decision-making [5-7]. For example, Wilson and Daly [5] and Van den Bergh *et al.* [4] suggested that males’ discounting rates rise after viewing images that evoke positive emotion. Similarly, during positive emotion, extraverted individuals were more likely to show preferences for immediate yet smaller reward over larger but delayed reward [7], while under negative emotional states people have tendencies to show more liking or favor more than usual without considering possibilities for a long-term effect [6]. This phenomenon of individuals’ seeking behaviors for sooner yet smaller reward over later but greater reward is called delay discounting, which also relates to one’s impetuous tendency or impulsiveness, wanting to receive an immediate reward. This temporal discounting of delayed rewards has been a widely used assessment tool for measuring impulsivity or impulsive decision-making [8].

Neurologically, the question is (1) how the brain functions under circumstances requiring an individual’s inter-temporal choice, and also (2) how myopic and forward-looking brain systems value rewards under the influence of emotion. In cognitive neuroscience, it is said that there are two distinct processes in inter-temporal decision-making. One is the valuation network, a neural representation of the subjective values of available decision options; the other is the cognitive control network, which comprises processes leading to and supporting action selection [9]. Peters and Büchel [10] suggest that inter-individual differences in delay discounting are likely a result from differences in the way information is processed in these neural systems. Inability to accurately represent the incentive value of future rewards (valuation deficit) and/or inability to exert top-down control over a disadvantageous choice tendency (self-control deficit) is an example.

Current research findings available provide an understanding of the neural mechanisms underlying delay discounting [9,11,12]. However, such findings are limited to accurately understand the specifics of neural activity, such as changes or differences in brain regions associated with inter-temporal choice under different emotional states. More studies are needed that meticulously explore neural activities that occur simultaneously with subjects’ inter-temporal decision-making under emotional changes, especially with visual presentation support using functional magnetic resonance imaging (fMRI) results.

This study examined the impact of emotion (both positive and negative) on subjects’ inter-temporal decision-making and investigated associated changes in brain activities in conjunction with arousal conditioning. During the delay-discounting task, neurological changes in brain activity were explored for each emotion manipulation, especially the basal ganglia receiving substantial reward-related information and the prefronto-parietal regions, previously suggested to be related to cognitive processes in evaluating future rewards and making numerical computation possible [12]. This study expected that high arousal induced by emotional stimuli will likely heighten subjects’ preferences for small but immediate reward than larger reward that is to come later, compared to the neutral conditioning, resulting in greater discounting of future larger rewards, indicative of increased impulsivity.

## Methods

### Participants

Twenty healthy male subjects (mean age of 23.95 years old; ranging 20 to 29 years old, right-handed) participated in the study. All subjects had normal or corrected-to-normal vision and no history of psychiatric diagnoses and neurological or metabolic illnesses. None of the subjects were taking any prescribed or over-the-counter medication on a regular basis at the time of the study. All subjects were unbiased and uncontaminated before and during the study. Informed consent was obtained from the subjects following a general description of the research protocol and were compensated for their voluntary participation. This study was approved by the Institutional Review Board at the Seoul National University (protocol # H-1006-059-321).

### Stimulus material

The stimulus materials specifically developed and tested consisted of 120 pictures representing three emotion categories: 40 high arousal-positive valence, 40 high arousal-negative valence, and 40 neutral pictures. For the experimentation, the researchers developed and tested a separate stimuli tool from the International Affective Picture System (IAPS) due to its inapplicability in effectively provoking positive and negative emotion in Korean male college students [13]. Affective pictures were partially drawn from the IAPS [14], then a unique stimulus material was developed as other studies previously have performed [15,16]. More specifically, in composing each stimuli condition, pictures that are known to provoke high arousal-negative valence and high arousal-positive valence were selectively chosen from IAPS [17]. Simultaneously, more photos were obtained from the Internet that contained images of most frequently used contexts in provoking emotion used in other similar research [15]. Images prepared for positive emotion included erotic (bodily) pictures depicting partially or completely naked women. Additionally, photos that were designed to evoke negative emotion included scenes showing bodily mutilation. Pictures used for the neutral emotional conditioning were specially created by systematically manipulating the pixels of both the negative and positive pictures into a colored ‘swirl’ so as to create distorted images with contents becoming unrecognizable from its original format, serving a comparison condition. Based on the arousal and valence scores that previous studies used [15,18], the images used in this study were all tested to verify whether or not the conditions successfully provoked high arousal-positive emotion and high arousal-negative emotion.

The selection of the emotion-inducing pictures was made in several steps. Three raters prechose 70 pictures for each of the three categories among 500 pictures collected from the internet and IAPS. One hundred male volunteers rated 210 pictures for valence and arousal dimensions on two nine-point visual analog scales, with one being the strongest negative valence and nine being the strongest positive valence, and then rating the degree of arousal with one being the least arousing and nine being the strongest arousing. Based on these valence and arousal ratings, the affective pictures were rank ordered, and the top 40 pictures for each valence dimension were selectively chosen. That is, the negative and positive images were chosen from the extremes of the arousal and valence dimensions so as to create two distinct conditions: high arousal-positive valence and high arousal-negative valence. Finally, another group of 32 participants (separate from the 100 volunteers who rated the pictures previously, ranking the 70 pictures for each valence and arousal dimension) rated these 120 selected pictures (top 40 pictures, each chosen for the total three emotional conditioning stimuli) for valence and emotional arousal level on the same two nine-point scales. Prior to the actual testing with the 20 subjects, the usefulness of these pictures was determined based on the preliminary statistical findings from the scores collected from the 32 participants to verify whether or not the stimuli served the originally intended purposes as conditional stimuli and discriminant from the neutral condition, as expected by the researchers.

The preliminary findings suggest that both positive and negative pictures were valid: positive pictures useful in truly capturing positive emotion and negative pictures also useful in capturing negative emotion (valence: negative = 1.94 ± .98, neutral = 4.62 ± 1.62, positive = 7.00 ± 0.98, *F* (1.31) = 47.62, *P* < .001; arousal: negative = 7.66 ± 1.23, neutral = 3.25 ± 1.63, positive = 7.06 ± 1.22, *F* (1.31) = 95.57, *P* < .001).

### Delay-discounting task

**Figure 1:**
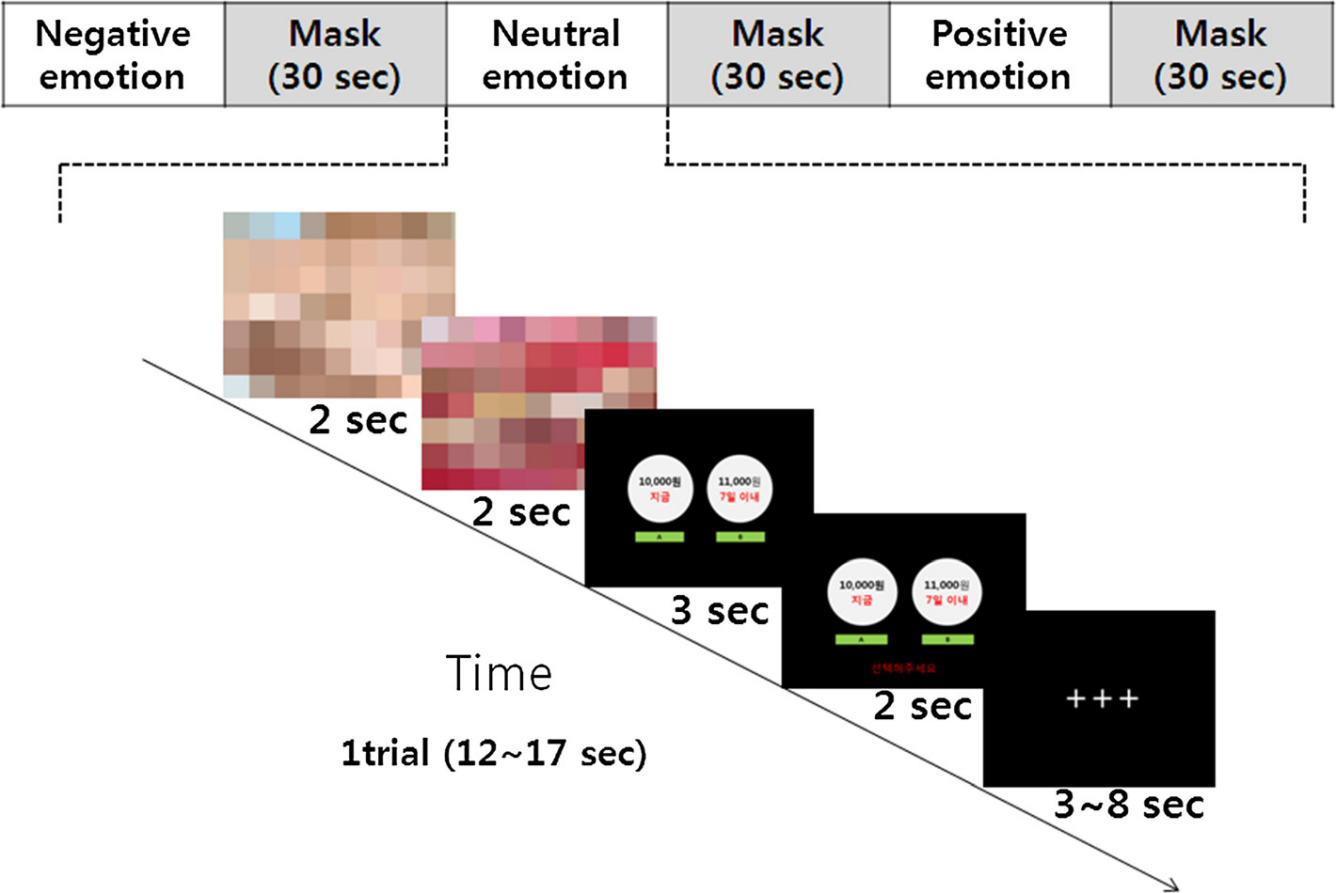
**Task design: participants performed delay-discounting task after affective picture presentation.**

During the fMRI run, subjects completed delay-discounting tasks via a computerized system (see Figure 1). Subjects were repeatedly presented with two optional choices with a numeric variance between a smaller but sooner reward and a larger yet delayed reward by pressing a corresponding button on a response box [19].

Subjects were asked to make a decision choosing between a smaller amount of money (that is, 10,000 won, equivalent USD $9) and any amounts greater than 10,000 won (for example, 11,000, 13,000, 15,000, 20,000, or 25,000 won) with a varying time frame (that is, 7, 30, 90, or 180 days). These choices were randomly displayed each time in either to the right or to the left of the response box for 3 s to avoid lateralized motor preparation in choosing either one of the two responses. In case subjects missed the opportunity to respond to the question within the first 3 s, they were given an additional 2 s to make the selection for the same question during this period. Others, who responded within the 3 s, waited during this same time period with a hold screen so they cannot make changes to the answer already chosen. Each trial ended with a 3- to 8-s variable inter-trial interval (ITI), giving a total of 12 to 17 s per trial period, so that participation time in total does not exceed 16 min. Subjects were informed that, after the experiment, they would receive a real payment of between 20,000 and 50,000 won in total for two randomly drawn amounts from their 60 responses (both considering the randomly selected amount and corresponding matching payment period) to ensure incentive-compatibility.

Estimation on individual participants’ degree of delay discounting was based on the formula *V* = *A*/(1 + *kD*), where *V* = subjective value of the delayed reward, *A* = full amount of the delayed reward, *k* = the rate at which delayed rewards are discounted, *D* = delay duration. Higher *k* values (discounting rates) indicate preferences for immediate rewards.

### Experimental procedures

Before beginning with the experiment, participants were given instructions about the experimental setup and protocol. Subjects were provided with an opportunity to participate in a trial of a practice session of the delay discounting task for at least 5 min. Participants were then led to the fMRI scanner room and positioned in the whole-body scanner, where they completed the actual experiment. All stimuli were back-projected onto a screen and viewed by participants through an angled mirror. One session was provided per subject, and one session consisted of a single run that included 60 trials. That is, the single run was made up of three emotional conditions (positive, negative, and neutral), yielding a total of 60 trials. Three types of pictures and delay-discounting tasks were presented to the participants in a single run. The order of the emotional conditions was counterbalanced for each participant to allow random conditioning of each task and to avoid order effect. Each trial presented two different pictures (for 4 s) and an instruction to passively view the pictures, and then participants performed delay-discounting tasks. E-Prime 2.0 (Psychology Software Tools, Inc., Pittsburgh, PA, USA) was used to present the stimuli and to collect the data. Immediately following the fMRI experiment, the participants viewed the same pictures again in the same order on a computer screen outside the scanner and were asked to complete two nine-point scales (developed for this research also used in the preliminary analysis on the validation of the usefulness of the pictures).

### fMRI Data acquisition and analysis

Using a 3.0-T magnetic resonance (MR) scanner (FORTE, ISOL Technology, Republic of Korea), the blood-oxygenation-level-dependent (BOLD) changes and structural MR images were obtained. Specifically, the BOLD response was measured using the echo planar imaging (EPI) sequence (TR = 3,000 ms, TE = 35 ms, flip angle 80°, 35 slices, slice thickness = 4 mm without gap, matrix size 64 × 64 mm, field of view (FOV) = 240 × 24 0 mm). T1-weighted fluid-attenuated inversion recovery (FLAIR) images (TR = 2,800 ms, TE = 16 ms, flip angle 60°, 35 slices, slice thickness = 4 mm, matrix size 256 × 256 mm, FOV = 240 × 240 mm) were also acquired. A total of 319 scans were recorded for each participant in a single session. At the start of each functional scanning run, the screen remained black for 9 s to allow time for magnetization to reach the steady state and to allow for the subjects’ adaptation to the situation. The first three images were discarded from the analyses.

An event-related fMRI design was used to identify activated brain regions. Analyses focused on changes in activation, specifically during the choice epochs (corresponding to the time between presentation of the choice and the subject’s recorded response). All image processing and analysis of this fMRI study was performed using SPM8 (Wellcome Department of Imaging Neuroscience, London, UK; http://www.fil.ion.ucl.ac.uk/spm) implemented in MATLAB 7.1. Individual scans were realigned and slice time corrected, normalized, and spatially smoothed by an 8-mm isotropic Gaussian kernel using standard SPM methods. The voxel dimensions of each reconstructed scan were 3 × 3 × 3 mm^3^ in the *x*, *y*, and *z* dimensions, respectively. Population inference was made through a two-stage procedure. At the first level, single-subject analysis was performed in the context of the general linear model (GLM), using delta functions convolved with the canonical hemodynamic responses to event during each condition. The six movement parameters from the realignment procedure were included in a general linear model as covariates. In a second-level analysis, a random-effect paired *t*-test analysis was performed to identify significantly different brain regions activated during positive or negative conditions in reference to those of the neutral condition to assess the significant effect at the group level. In order to provide a focused test of our hypothesis, analysis employed a region of interest (ROI) approach. Based on an extensive literature review, the regions of interest were defined, which included the basal ganglia, the prefrontal cortex, and the parietal cortex in both hemispheres. Subsequently, MarsBaR software (http://marsbar.sourceforge.net) was used to extract the percent signal changes of the activated regions. The determination of the ROIs was made by drawing a circle of 5 mm in radius based on the voxel of activated cluster in the study among the selected ROIs from the previous literatures. In agreement with previous studies, we used a threshold of *P* < 0.001 uncorrected, rather than the more rigorous *P* < 0.05 corrected for the entire brain volume [20]. An extended threshold of 50 contiguous voxels was then applied to the activation.

## Results

### Self-reported emotion and task performance

Subjective ratings on emotional images and behavioral data are shown in Table 1. Repeated ANOVA was used to confirm the valence and arousal dimension of the positive, negative, and neutral emotion. Findings show that positive stimuli increased self-reported positive valence (7.05 ± 0.94) and negative stimuli increased self-reported negative valence (1.75 ± 0.85), in reference to neutral-stimulus-induced valence (4.25 ± 0.85, *F* (1.19) = 72.31, *P* < .001). Additionally, positive and negative stimuli increased self-reported arousal (7.15 ± 1.26, 7.60 ± 1.53), relative to neutral-stimulus-induced arousal (3.25 ± 1.65, *F* (1.19) = 49.91, *P* < .001). Behavioral analyses showed that discount rates were significantly lower during the neutral condition (0.0036 ± 0.0036), compared to the positive and negative condition (0.0045 ± 0.0033 and 0.0051 ± 0.0048, respectively). This result indicated that subjects’ choice behavior was more impulsive during the positive and negative condition, compared to the neutral condition (*F* (1.19) = 6.91, *P* < .05).

**Table 1:** **Self-reported emotional dimension and behavioral results**

		**M (SD)**			***F***
		**Negative**	**Neutral**	**Positive**	
Self-reported emotion	Valence arousal	1.75(0.85)	4.25(0.85)	7.05(0.94)	72.32***
		7.60(1.53)	3.25(1.65)	7.15(1.26)	49.91***
Delay-discounting task	*k*	0.00045	0.0036	0.0051	6.91*
		(0.0033)	(0.0036)	(0.0048)	

*P < 0.05, ***P < 0.001. Note: M (SD) represents means (standard deviations).

### fMRI results

**Figure 2:**
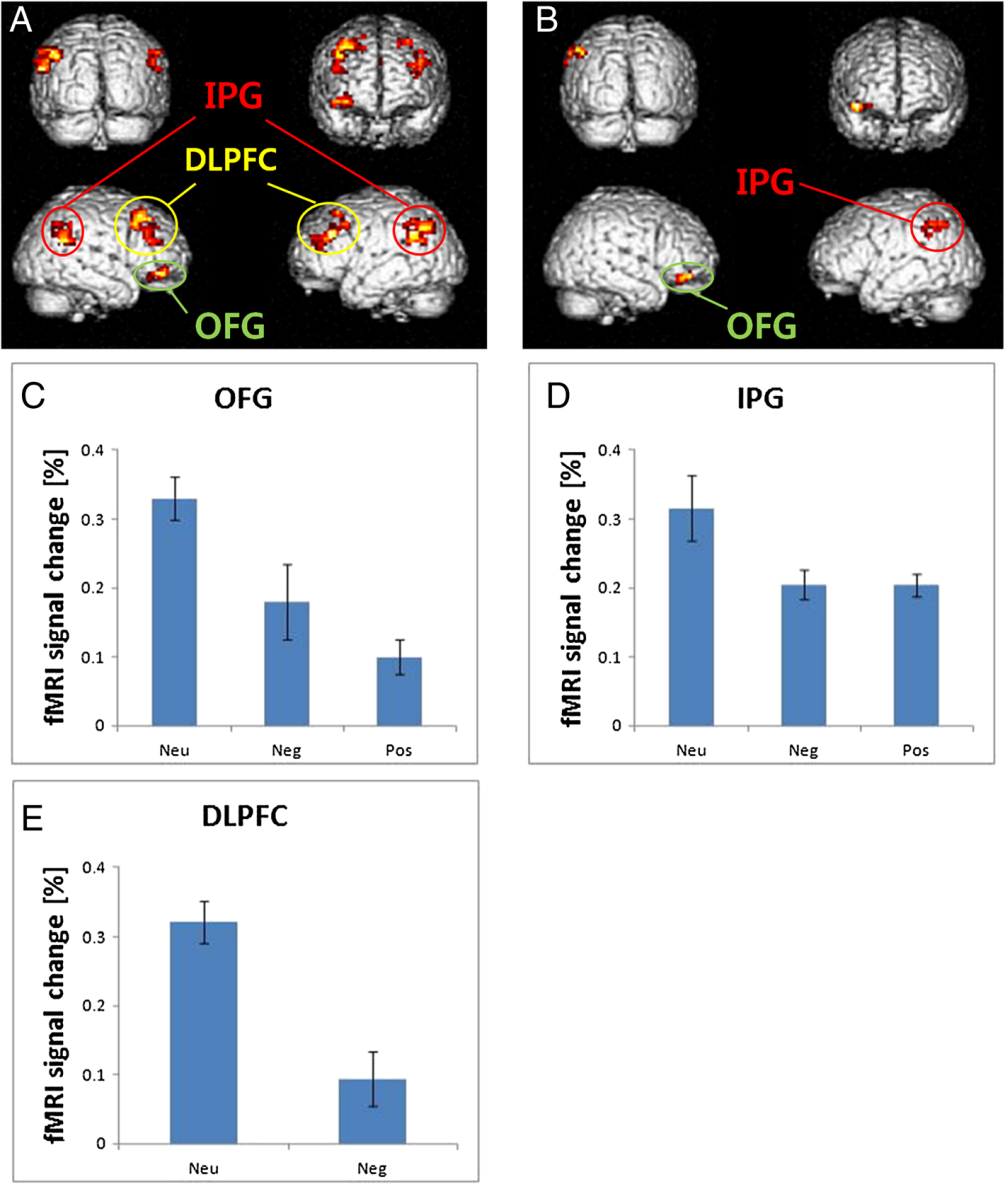
**Neuroimaging results: brain regions activated in delay discounting task. (A)** Neutral minus negative emotional condition. **(B)** Neutral minus positive emotional condition. **(C, D, E)** Shown are the mean activations of the orbitofrontal gyrus (OFG), inferior parietal gyrus (IPG), and dorsalateral prefrontal cortex (DLPFC) expressed as percent signal change. In these regions, activation was greatest in the neutral condition. *Neu* neutral, *Neg* negative, *Pos* positive.

Analysis on the BOLD response for the delay discounting following emotional manipulation was conducted by calculating the following four contrasts: neutral minus negative (neu > neg), neutral minus positive (neu > pos), negative minus neutral (neg > neu), and positive minus neutrals (pos > neu). The ROIs within group analysis are illustrated in Table 2 and Figure 2, with their *x-*, *y-*, and *z*-coordinates and cluster sizes.

**Table 2:** **Statistical regions of interest**

				**Talairach coordinates (mm)**		
	**Brain region**	**BA**			**t_value**	**z_value**
				***x*** **,** ***y*** **,** ***z***		
Neu > Neg	(L) inferior parietal gyrus	39	260	−42, −63, 39	10.6	5.99
				−45, −51, 30	7.43	5.03
				−54, −60, 24	7.33	4.99
				−33, 30, 27	8.63	5.44
	(L) dorsalateral prefrontal cortex	9	306	−27, 24, 51	7.44	5.03
				−39, 30, 33	7.38	5.01
				42, 18, 42	8.41	5.37
	(R) dorsalateral prefrontal cortex	8	311	18, 30, 45	7.87	5.19
				30 24 51	7.82	5.19
				36, 48, −12	8.35	5.35
	(R) orbitofrontal gyrus	11	91	45, 33, −15	5.55	4.23
				51, −51, 33	6.98	4.86
	(R) inferior parietal gyrus	40	156	51, −48, 42	6.55	4.68
				60, −48, 42	6.4	4.62
Neu > Pos	(R) orbitfrontal gyrus	11	60	42, 39, −12	6.88	4.82
				27, 48, −12	4.27	3.53
				−48, −57, 45	6.12	4.49
	(L) inferior parietal gyrus	40	78	−54, −45, 42	4.91	3.9
				−60, −48, 33	4.4	3.61

Note: BA represents Brodmann area; Neu, Neg, and Pos represent neutral, negative, and positive, respectively.

#### Neu > Neg

During the neutral condition, compared to the negative condition, greater activation was detected in the bilateral inferior parietal gyrus (IPG, Brodmann Area (BA) 39/40), the bilateral dorsalateral prefrontal cortex (DLPFC, BA 8/9), and the right orbitofrontal gyrus (OFG, BA 11) (Figure 2A).

#### Neu. > Pos.

Similar to the results of neutral minus negative condition, the differences in brain activity for the contrast neutral minus positive condition was identified in the right OFG (BA 11) and the left IPG (BA 40) (Figure 2B).

#### Neg > Neu Pos > Neu

There were no brain areas that were more active during the negative and positive condition compared to the neutral condition.

## Discussion

This study examined the impact of emotion on subjects’ inter-temporal decision-making. Behavioral results showed that delay discounting is likely to be influenced by the ‘high arousal-positive valence’ and ‘high arousal-negative valence’ emotion. This indicates that, regardless of positive emotion or negative emotion, increased impulsive decision-making occurs under high-arousal emotional state, compared to the conditioning of the neutral state. These findings are consistent with previous studies such as in Peters *et al.* [21] that identified a relationship between greater impulsive decision-making and emotional arousal. For example, Ariely and Loewenstein [22] found that objects perceived as relatively unattractive in a non-aroused state became attractive substances during an aroused state. Herman and Polivy [23] also emphasized the role of emotional arousal on increasing cognitive disruption in making consistent decisions among addicts and dieters. They suggested that emotional arousal experience can lead to a motivational shift in individuals’ decision-making to seek immediate satisfaction over long-term goals.

The brain imaging data of this study also illustrated that brain activity associated with inter-temporal decision-making can substantially change by a certain state of arousal. More specifically, decision-making following exposure to neutral emotion was positively associated with activity in prefronto-parietal regions, while decision-making following exposure to high arousal positive/negative emotion was adversely associated with activations in DLPFC, OFG, and IPG. Different from our expectation, we could not find the activation of the basal ganglia; this might be due to the insensitive parameter that we used. That is, increased impulsive decision-making is related to situational high arousal (that is, visceral factors) and decreased activities in the prefronto-parietal regions, brain regions responsible for a human’s decision-making processes associated with the deliberative system as suggested by the dual-system perspective on human cognition. These findings indicate that people tend to make impulsive decisions during emotionally aroused states than the state of non-arousal [21], because the deliberative system’s ability to exercise regulatory control over impulsive behavior becomes functional when individuals are not emotionally excited.

This prefronto-parietal region is also known to play an important role in explaining the neural mechanisms of inter-temporal decision-making, which makes delayed choices with greater reward possible over immediate options [11,24]. As mentioned previously, Peters and Büchel [10] suggest that valuation network (that is, dual valuation system and single valuation system) and cognitive control network play an important role in inter-temporal decision-making. In particular, choosing larger yet delayed rewards were associated with increased activity in the prefronto-parietal regions, and this finding is consistent partially with previous research that suggests the dual valuation system in the valuation network [11,24]. The dual valuation system explains the discrepancy between the short-run and long-run preferences, reflecting the differential activations of distinguishable neural systems (so-called β-δ model). Simply put, the idea is that when people are faced with a choice between a small but immediate reward and a larger yet delayed reward, choosing the latter is associated with decreased activity in the β network including limbic brain regions and increased activity in the δ network including the lateral orbitofrontal cortex (LOFC) and the dorsolateral (DLPFC) and ventrolateral (VLPFC) portions of the prefrontal cortex and the posterior parietal cortex (PPC). This study’s findings, therefore, can be interpreted that the decreased activity in prefronto-parietal regions during the high-arousal states results in the inability to accurately represent the value of future rewards.

In addition to these effects of the valuation system, decision-making is also said to be affected by the cognitive control network. Opting for a larger yet delayed reward requires cognitive control to precisely evaluate the trade-offs among outcomes occurring at different points in time, preventing from making automatic responses seeking immediate rewards, and thus, making longer delays elicit activation in cortical regions involved in cognitive control [11,24]. One function of the prefrontal cortex (PFC) during delay discounting is the exertion of cognitive control to overrule short-sighted choices [25]. As Peters and Büchel [10] suggest, inter-individual differences in the degree of PFC recruitment contribute to inter-individual variability in discounting, with greater PFC involvement probably reflecting enhanced cognitive control and, as a result, less impulsive decision-making. In this study, PFC was significantly less activated when making decisions during high arousal-positive and high arousal-negative emotion states than in the neutral emotion condition. These findings indicate that high arousal inhibits individuals’ exerting top-down cognitive control of the PFC.

This study offers meaningful information to the current literature. This is the first fMRI study to investigate and identify neural activity that accounts for the impact of induced incidental arousal on inter-temporal decision-making. Implications of this study include broadening our understanding on the impact of the high-arousal state on the increased likelihood of individuals’ preferring smaller and earlier rewards, as opposed to waiting for larger rewards that is to come later. This is explained by high arousal reducing our cognitive consideration for the future as well as inhibition of cognitive control. These results may explain the fundamental reason why irrational decisions are made and less accurate problem solving is performed during periods of intense emotion, compared to periods of mild emotion or relaxation [1]. This emphasizes the need for ‘stabilization of high emotional arousal’ in attempts to facilitate a reduction of impulsive tendency, maintenance of self-control, and implementation of longer term goals. The study findings may be useful in providing valuable information for assisting clinical interventions in which treatment goals are to reduce impulsivity or impulsive decision-making, such as self-regulation strategies using meditation that are used to facilitate relaxation.

This study, however, is not exempt from limitations, such as the focus being on young healthy male college students, which may limit the generalizability of the study findings to the overall population, especially for people with limited cognitive functioning. Future research is suggested to broaden the sample selections considering diverse age, gender, and psychological health statuses that may exhibit differences in the way people react to stimuli and behave under emotional states and compare with the current findings to verify the applicability of the experiment conditions as well as key findings. Future research may also consider exploring differences in decision-making and brain activity associated with different levels of arousal, that is, high and low, or different emotions that are within the same valence dimension, such as sadness and anxiety, as Lerner and Keltner [26] suggest. Incorporation of this aspect by distinguishing the level of arousal for the experiment conditioning, that is, high arousal-positive valence (for example, joy), low arousal-positive valence (for example, calmness), high arousal-negative valence (for example, fear), and low arousal-negative valence (for example, sadness) may add greater precision in determining the effects of emotion on inter-temporal decision-making and brain activity.
